# Effects of stimulant drugs on actual and simulated driving: perspectives from four experimental studies conducted as part of the DRUID research consortium

**DOI:** 10.1007/s00213-012-2766-1

**Published:** 2012-06-15

**Authors:** J. G. Ramaekers, K. P. C. Kuypers, W. M. Bosker, K. A. Brookhuis, J. A. Veldstra, R. Simons, M. Martens, M. Hjälmdahl, Å. Forsman, A. Knoche

**Affiliations:** 1 Department Neuropsychology and Psychopharmacology, Faculty of Psychology and Neuroscience, Maastricht University, Maastricht, The Netherlands; 2 University of Groningen, Groningen, The Netherlands; 3 Delft University of Technology, Delft, The Netherlands; 4 TNO Human Factors, Soesterberg, The Netherlands; 5 VTI, Linköping, Sweden; 6 BASt, Bergisch Gladbach, Germany

## Commentary

The Integrated Project DRUID (Driving under the Influence of Drugs, Alcohol and Medicines) involved researchers from more than 20 European countries. It aimed to gain new insights to the degree of impairment caused by psychoactive drugs and their actual impact on road safety. Part of this large research program that was conducted between 2006 and 2011 has been devoted to the assessment of stimulant drug effects on driving performance in experimental, placebo-controlled studies. These studies are presented in the current issue of psychopharmacology and focus on single-dose effects of MDMA (Bosker et al. 2012) and dexamphetamine (Hjalmdahl et al. 2012) on driving performance before and after a night of sleep deprivation and on the effects of MDMA (Veldstra et al. 2012) and dexamphetamine (Simons et al. 2012) with and without alcohol. The major objective of these studies was to provide scientific basis for harmonized pan-European regulations of driving under the influence (DUI) of stimulants. Research partners agreed on a number of standardized driving scenarios to increase comparability between studies. These included a road tracking test to measure standard deviation of lateral position (SDLP) or “weaving motion” during prolonged highway driving (O'Hanlon et al. 1982), a car-following scenario to measure a driver's ability to adapt to maneuvers of other motorists (Brookhuis and de Waard 1993; Ramaekers and O'Hanlon 1994), and in case of driving simulator studies, risk-taking scenarios. In addition, all partners included a number of laboratory tests measuring skills related to driving. These tests included tracking tasks, attention tasks, reaction tasks, and cognitive tasks. Significant drug effects were statistically evaluated for clinical relevance by equivalence testing. Equivalence testing of drug effects was based on difference in scores from placebo relative to an alcohol criterion (i.e., equivalence to a blood alcohol concentration (BAC) of 0.5 mg/mL). Basically, equivalence testing assessed whether the alcohol criterion value falls within the 95 % confidence interval (CI) for the drug effect. If yes, then the drug effect was considered equivalent to a BAC of 0.05 mg/mL (and thus clinically relevant for traffic safety). If the 95 % CI was below the alcohol criterion value, then a drug effect was considered not relevant. An integrative overview of results from the four experimental studies is presented in Tables 1 and 2.

**Table 1:** Summary of MDMA and dexamphetamine effects on primary and secondary driving parameters (improvement, neutral, or impairment), as well as subjective measures of arousal or sleep

	MDMA–sleep deprivation study			Dexamphetamine–sleep deprivation study		
	(Bosker et al. 2012)			(Hjalmdahl et al. 2012)		
	MDMA	Sleep deprivation	MDMA + sleep deprivation	Dexamphetamine	Sleep deprivation	Dexamphetamine + sleep deprivation
Road tracking	No effect^a^	Increased SDLP^b^	Increased SDLP^b^	No effect^a^	Increased SDLP^b^	Increased SDLP^c^
		Impairment > BAC 0.8 mg/mL^b^	Impairment > BAC 0.8 mg/mL^b^		Impairment > BAC 0.5 mg/mL SDLP^b^	Relevance of impairment undecided (95 % CI drug effect includes BAC 0.5 mg/mL as well as 0)^c^
Car following	No effect^a^	No effect^a^	No effect^a^	Dose-related improvement of phase delay^a^	Impairment of phase delay^b^	Impairment of phase delay^b^
Risk taking	Not assessed	Not assessed	Not assessed	Improvement RT to crossing cars^a^	Improvement RT to crossing cars^a^	Improvement RT to crossing cars^a^
Laboratory measures of skills related to driving	Neutral on most measures^a^	Impairment of attention and impulse control^b^	Impairment of attention and impulse control^b^	Not assessed	Not assessed	Not assessed
	Improvement on rapid information processing^a^					
Subjective measures	Increased arousal^a^	Decreased arousal^b^	Decreased arousal^b^	Decreased sleepiness^a^	Increased sleepiness^b^	Increased sleepiness^b^

*RT* reaction time

^a^Neutral effects or “stimulating effects”

^b^“Impairing” effects

^c^Impairments associated with a wide 95 % CI, which indicate a large variety in response; some subjects are as impaired as under alcohol, and others perform as under placebo

**Table 2:** Summary of MDMA and dexamphetamine effects on primary and secondary driving parameters (improvement, no effect, or impairment) as well as subjective measures of arousal and sleep, alone, and in combination with alcohol

	MDMA–alcohol study			Dexamphetamine–alcohol study		
	(Veldstra et al. 2012)			(Simons et al. 2012)		
	MDMA	Alcohol	MDMA + alcohol	Dexamphetamine	Alcohol	Dexamphetamine + alcohol
Road tracking	Decrease SDLP^a^	Increase SDLP^b^	Increase SDLP^c^	No effects^a^	Increased SDLP^b^	Increased SDLP^c^
			Relevance of impairment undecided (95 % CI drug effect includes BAC 0.5 mg/mL as well as 0)^c^			Relevance of impairment undecided (95 % CI drug effect includes BAC 0.8 mg/mL as well as 0)^c^
Car following	No effect^a^	No effect^a^	No effect^a^	No effect^a^	No effect^a^	No effect^a^
Risk taking	No effect^a^	No effect^a^	No effect^a^	No effect^a^	Shorter gap acceptance; increased red light crossings and number of crashes^b^	Shorter gap acceptance; increased red light crossings and number of crashes^c^
						Relevance of impairment undecided (95 % CI drug effect includes BAC 0.8 mg/mL as well as 0)^c^
Laboratory measures of skills related to driving	Not assessed	Not assessed	Not assessed	No effect^a^	Impairment of attention, tracking, and RT^b^	Impairment of attention, tracking, and RT^b^
Subjective measures	Decreased sleepiness^a^	Increased sleepiness^b^	Increased sleepiness^b^	Decreased sleepiness^a^	No effect^a^	Decreased sleepiness^a^

*RT* reaction time

^a^Neutral effects or “stimulating effects”

^b^“Impairing” effects

^c^Impairments associated with a wide 95 % CI, which indicate a large variety in response; some subjects are as impaired as under alcohol, and others perform as under placebo

### Effects of MDMA and dexamphetamine

The effects of MDMA and dexamphetamine on measures of simulated and actual driving were neutral for most of the driving measures or even positive for specific measures (i.e., road tracking). Stimulatory effects were also supported by subjective data that indicated that MDMA and dexamphetamine increased arousal and decreased sleepiness. The stimulatory effects of stimulants on human performance have been widely acknowledged (Ramaekers et al. 2006; Kuypers et al. 2006), and as such are no real surprise. It should be noted, however, that previous research had also demonstrated that stimulant drugs can improve certain aspects of performance while impairing other performance domains at the same time. For example, stimulants have repeatedly been shown to improve neuropsychological skills, such as tracking, impulse control, and reaction time, while impairing cognitive functions such as working memory and movement perception (Kuypers and Ramaekers 2005; Lamers et al. 2003; Silber et al. 2006, 2005; Ramaekers et al. 2009). Thus, the finding that MDMA and dexamphetamine can improve performance in particular driving domains does not automatically mean that these drugs do never have detrimental effects in other domains relevant to driving as well.

### Effects of alcohol alone and combination with MDMA and dexamphetamine

Alcohol was administered in two simulated driving studies in order to assess the potential interaction between alcohol–MDMA and alcohol–dexamphetamine. Alcohol significantly impaired road tracking performance in the study by Simons et al. (2012). Coadministration of dexamphetamine did not significantly change the impairing effect of alcohol as evinced by the lack of statistical interaction between dexamphetamine and alcohol. Equivalence testing demonstrated that the 95 % CI of the change in road tracking performance (i.e., SDLP) after combined use of dexamphetamine and alcohol included both the alcohol criterion as well as the placebo reference (zero). The latter basically means that the evaluation of the clinical relevance of the combined effects of dexamphetamine and alcohol are undecided or ambiguous, i.e., it is predicted that some individuals will show impairment, whereas others may not.

Risk scenarios and secondary driving measures employed by Simons et al. (2012) were very sensitive to the effects of alcohol alone and to alcohol–dexamphetamine combined. These measures demonstrated that single doses of alcohol (0.8 g/kg body weight) increased risk-taking behaviors (i.e., shorter gap acceptance, increase of red light crossings, and number of crashes) and impaired tracking, attention, and reaction time during a 3-h period after drinking when BACs declined from 0.9 to 0.2 mg/mL. Moreover, these alcohol impairments were not affected by the coadministration of dexamphetamine 20 mg, indicating that the stimulatory effects of dexamphetamine were not sufficient to overcome the impairing effects of alcohol on skills related to driving.

Alcohol effects were most prominent in the road tracking scenario in the MDMA–alcohol interaction study conducted by Veldstra et al. (2012). As expected, alcohol significantly increased SDLP or the amount of “weaving” during highway driving, suggesting a decrement of road tracking control. This finding nicely replicates earlier demonstration of alcohol-induced impairment of road tracking performance in actual, on-the-road driving test scenarios (Kuypers et al. 2006; Louwerens et al. 1987). Average BACs during the simulated driving tests were around 0.45–0.50 mg/mL during treatments with alcohol. The stimulatory effects of MDMA (100 mg) were sufficient to counteract some of the impairing effects of this low dose of alcohol on SDLP as indicated by a significant MDMA × alcohol interaction. However, equivalence tests again demonstrated that change in SDLP after the combination of MDMA and alcohol included both the alcohol criterion as well as the placebo reference. In other words, due to large variation in subject sensitivity to combination of MDMA and alcohol, some subjects showed impairment, whereas others did not. These findings are in line with previous research that also indicated that stimulatory effects of MDMA are not sufficient to fully overcome alcohol-induced impairments of driving performance, psychomotor function, and cognition (Kuypers et al. 2006; Brookhuis et al. 2004; Dumont et al. 2008; Kuypers et al. 2006; Ramaekers and Kuypers 2006; Hernandez-Lopez et al. 2002).

### Effects of sleep deprivation with or without MDMA or dexamphetamine

The sleep deprivation studies demonstrated that sleep loss produced severe impairment in actual and simulated driving performance as expressed by a significant rise in SDLP in the road tracking scenario. In the on-the-road driving study (Bosker et al. 2012), a large number of driving tests were prematurely terminated due to excessive fatigue.

On average, SDLP increased with 4.2 cm in the morning after sleep deprivation, relative to SDLP before sleep deprivation. This increment is about 1.5–2 times greater than that found in two recent driving under the influence of alcohol studies with blood alcohol concentrations between 0.29 and 0.5 mg/mL (Kuypers et al. 2006). From a previous alcohol study that was conducted in order to calibrate SDLP for the dose-related effects of alcohol (Louwerens et al. 1987), it can be concluded that a mean increase in SDLP of 4.2 cm is equivalent to a blood alcohol concentration of approximately 0.8 mg/mL. Equivalence testing even demonstrated that the upper limit of the 95 % CI associated with the mean change in SDLP after sleep deprivation widely exceeded the criterion level of 1.0 mg/mL BAC. These findings were corroborated by results from secondary driving measures as measured in laboratory tests. Critical tracking performance significantly decreased over the night, as a function of hours of sleep loss. Together, this indicates that sleep deprivation caused severe driving impairment comparable to driving under the influence of high to very high BAC.

It is also apparent from the present studies that the stimulant effects of MDMA and dexamphetamine, if any, could not compensate for the impairing effect of sleep loss on simulated and actual driving performance. None of the primary driving measures demonstrated any significant MDMA × sleep loss interaction. The effects of sleep deprivation on driving were highly prominent during MDMA and dexamphetamine treatments and did not change as a function of dose and concentration.

### Concentration effect relations of MDMA and dexamphetamine

Two studies were specifically designed to assess driving performance across a wide range of doses and concentrations. The MDMA–sleep deprivation study (Bosker et al. 2012) included three doses of MDMA (25, 50, and 100 mg), whereas the dexamphetamine–sleep deprivation study included a low dose (10 mg) and a high dose (40 mg) of dexamphetamine (Hjalmdahl et al. 2012). It was apparent in both studies that neither MDMA nor dexamphetamine produced any dose- or concentration-related effects on driving. Also, the inability of both stimulants to compensate for the impairing effects of alcohol and sleep deprivation was not affected by dose or concentration.

It should, however, be noted that doses administered in the present studies may have been relatively low for (some) recreational drug users. Dexamphetamine doses (10–40 mg) were well with the accepted therapeutic window when used for medical purposes. Likewise, MDMA doses were close to the amount of MDMA that is generally present in a single ecstasy tablet (i.e., 50–100 mg). However, it is very likely that a significant proportion of recreational drug users will take much higher doses of dexamphetamine and MDMA in real-life situations. High-dose effects of stimulant on driving performance cannot be readily assessed in experimental, placebo-controlled studies due to obvious medical and ethical constraints. It has become evident, however, that MDMA and amphetamine concentrations that are observed in actual DUI cases can be 10-fold higher than during controlled administration in experimental studies. A recent study analyzing drug concentrations in postmortem cases and DUI cases in the Netherlands in 1999–2004 may serve to illustrate this point (Verschraagen et al. 2007). Amphetamine-based drugs were present in 70 postmortem cases and 467 DUI cases. The most detected amphetamine-based drug was MDMA, followed by amphetamine. Median blood concentrations of MDMA in postmortem and DUI cases were 1,600 and 330 ng/mL, respectively. MDMA blood concentrations in the MDMA-related deaths (*n* = 20) and in the DUI cases (*n* = 360) varied up to 3,700 and 4,000 ng/mL, respectively. The median concentrations of amphetamine in the amphetamine-related deaths (*n* = 13) and the DUI cases (*n* = 208) were 280 and 220 ng/mL, respectively. Amphetamine blood concentrations up to 6,000 and 2,300 ng/mL were seen in the drug-related deaths and DUI cases, respectively. The most frequently encountered amphetamine-based drugs in the investigated deaths were MDMA and amphetamine. The majority of MDMA- and amphetamine-caused deaths, i.e., 90 % of these deaths, occurred with blood concentrations above 1,500 and 800 ng/mL, respectively. Clearly, these data show that amphetamine concentrations in DUI cases can be much higher than amphetamine concentrations that are achieved in controlled studies.

### MDMA, dexamphetamine, and driving safety

The pharmacological effects of stimulants and drug use settings seem very much intertwined and are likely to play a crucial role when evaluating driving under the influence offenders. Some will take the present data as an argument to show that the primary reason for impairment observed in DUI cases with stimulants will be sleep deprivation or concomitant use of alcohol and drugs. Others can argue that the use of stimulants may affect a person's ability to subjectively evaluate or recognize their state of impairment. Stimulants increase subjective feelings of arousal, energy, and mood. Such feelings affect the subjective judgment of stimulant users on whether or not it is safe to drive home after spending a night at a rave party. During intoxication with a stimulant, they may not be able to subjectively experience the debilitating effects of sleep loss or concomitant alcohol use to the same degree as stimulant-free drivers, because they feel more energetic. As a consequence, they may decide to drive because they subjectively feel alert while neglecting the objectively impairing effects of other cofactors, such as sleep deprivation or alcohol use.

In the context of a pan-European initiative to combat driving under the influence of drugs, it is advised to distinguish between (1) potential medicinal use of amphetamines (therapeutic doses) and (2) drug abuse of stimulants (polypharmacy and combat of sleep). Stimulants are generally safe for driving when taken alone at regular doses (e.g., as in medicinal use), but stimulant effects are less safe when taken in combination with sleep loss or alcohol intoxication as is often the case in drug abusers. In such cases, it will be very difficult to separate stimulant effects from those of drug use setting. Consequently, drivers should receive specific warnings on driving impairment arising from the use of stimulants during sleep loss or alcohol intoxication.
